# Bit-Related Lesions in Event Horses After a Cross-Country Test

**DOI:** 10.3389/fvets.2021.651160

**Published:** 2021-03-31

**Authors:** Kati Tuomola, Nina Mäki-Kihniä, Anna Valros, Anna Mykkänen, Minna Kujala-Wirth

**Affiliations:** ^1^Department of Production Animal Medicine, Research Centre for Animal Welfare, University of Helsinki, Helsinki, Finland; ^2^Independent Researcher, Pori, Finland; ^3^Department of Equine and Small Animal Medicine, Faculty of Veterinary Medicine, University of Helsinki, Helsinki, Finland; ^4^Department of Production Animal Medicine, Faculty of Veterinary Medicine, University of Helsinki, Helsinki, Finland

**Keywords:** animal welfare, eventing, Ulcer, BIT, horse

## Abstract

Bit-related oral lesions are common and may impair horse welfare. The aim of this study was to investigate the prevalence of oral lesions and their risk factors in a sample of Finnish event horses. The rostral part of the oral cavity (the bit area) of 208 event horses (127 warmbloods, 52 coldbloods, and 29 ponies) was examined in a voluntary inspection after the last competition phase, i.e., the cross-country test. Acute lesions were observed in 52% (109/208) of the horses. The lesion status was graded as no acute lesions for 48% (99/208), mild for 22% (45/208), moderate for 26% (55/208) and severe for 4% (9/208) of the horses. The inner lip commissure was the most common lesion location observed in 39% (81/208) of the horses. A multivariable logistic regression model with data of 174 horses was applied to risk factor analysis. Horses wearing thin (10–13 mm) (OR 3.5, CI 1.4–8.7) or thick (18–22 mm) (OR 3.4, CI 1.4–8.0) bits had a higher risk of moderate/severe lesion status than horses wearing middle-sized (14–17 mm) bits (*P* = 0.003). Breed was associated with moderate/severe lesion status (*P* = 0.02). The risk was higher for warmbloods (reference group) and coldbloods (OR 2.0, CI 0.88–4.7) compared with ponies (OR 0.2, CI 0.04–0.87). Mares were at higher risk of moderate/severe lesion status (OR 2.2, CI 1.1–4.5) than geldings (reference group) (*P* = 0.03). Bar lesions were more common in horses with unjointed bits (40%, 8/20) than with basic double-jointed (10%, 5/52), formed double-jointed (8%, 6/78) or single-jointed bits (5%, 2/40) (Fisher's exact test, *P* = 0.002). The results of this study suggest that thin and thick bits and mare sex should be considered risk factors for mouth lesions. In addition, in this sample ponies had smaller risk for lesions than other horse breeds. We encourage adopting bit area monitoring as a new routine by horse handlers and as a welfare measure by competition organizers for randomly drawn horses.

## Introduction

Horse welfare in competitive equestrian sports is under increasing scrutiny (1, 2), with attention being directed among others to bit-related lesions, which affect horse welfare by potentially causing anxiety, fear, and pain (3). Oral tissues have a strong somatosensory innervation (4, 5). The function of the nociceptive system is to detect potentially noxious mechanical, chemical, or thermal stimuli (4, 5). From animal welfare point of view it is essential to minimize such negative experiences by preventing or at least rapidly diagnosing and treating injuries and ensuring conditions which avoid suffering (6–8). Bit-related lesions in equestrian competitions have previously been examined in Icelandic horses, polo ponies, racehorses, riding horses and trotters (9–14). Curb bits with ports in Icelandic horses and Crescendo bits, unjointed regulator mullen mouth bits and straight plastic bits in trotters have been associated with higher lesion risk (9, 15). Thoroughbred racehorses with snaffle bits had multiple and more severe lesions than polo ponies wearing gag bits (12). Harness racing increased mucosal injuries in the rostral oral cavity compared to training (14). One study investigated event horses after a competition, but only the mouth corners were examined to detect lesions, because the rules of the governing equestrian federation did not allow for a full intraoral examination (13). Mouth corner lesions were present in six (7.5%) out of 80 event horses and in one (3%) out of 33 event ponies (13).

Although oral lesions are a commonly reported problem (9–12, 16–19) and of long-established citizen and veterinary concern (8, 19, 20), the underlying risk factors are not adequately described in scientific literature. The aim of this study was to analyze the occurrence of oral lesions in a sample of Finnish event horses and the potential association of lesions with equipment, such as noseband, bit type and bit thickness or other factors such as breed, age, sex, competition level or competition performance.

## Materials and Methods

### Horses and Oral Examination

Examinations of rostral mouth area were carried out in eight competition events at three locations in western Finland during the summers of 2018 and 2019. Apart from one international event all were national level competition events. The study was executed in collaboration with The Equestrian Federation of Finland (Suomen Ratsastajainliitto, SRL). The examination was voluntary. Information regarding the study was provided to participants when they registered online for the competition and in an information leaflet distributed on the 1st day when checking in at the competition office. All competitions were 2-day events. Dressage and show jumping phases were held on the 1st day. Examination was carried out on the 2nd day, after competitors had completed the cross-country test and approached the horse trailer area. An invitation to participate was extended to as many competitors as possible to maximize the sample size but horses examined in previous competitions were excluded. The majority of those approached (215/227, 95%) agreed to participate, and 97% of their horses (208/215) were successfully examined, with seven horses excluded due to obvious avoidance behavior during examination. Examination was performed from the left and right sides of the horse without sedation or mouth speculum, and with disposable nitrile gloves and an efficient headlamp (Lumonite Navigator 3,000 headlamp set at 420–1,300 lumens) (11) by a single veterinarian experienced in the oral examinations of horses. Lesions were assessed visually, and bars of the mandible, the area mesial to second lower premolar teeth, were also palpated. An assistant verified and recorded all the findings on an examination form. Examples of lesions were documented with a digital camera (Panasonic DMC-GX7; lens H-FS14140, 14–140) with the rider's permission.

**Figure 1:**
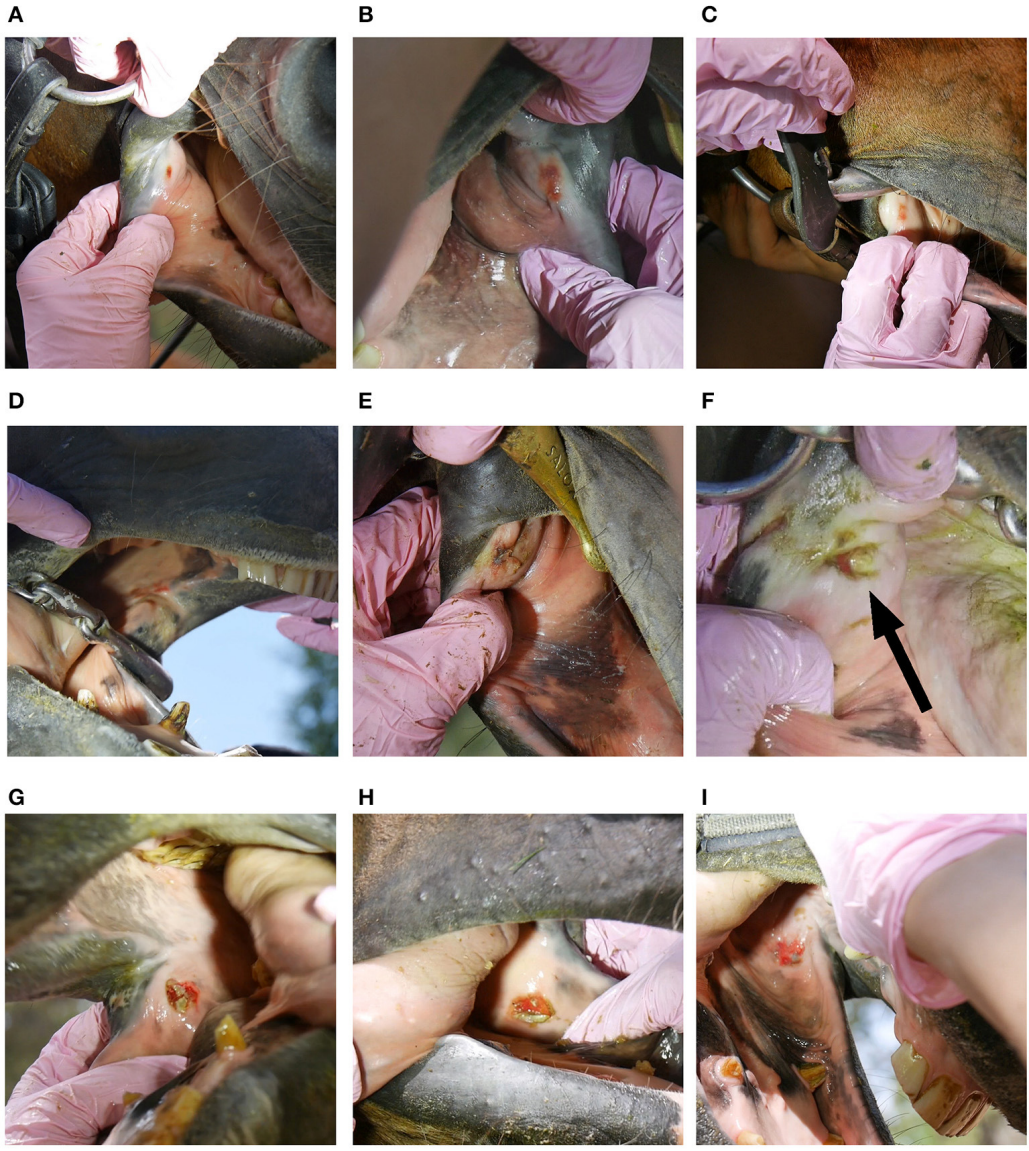
Bit-related inner lip commissure lesions; **(A)** bruise 2 points **(B)** bruise 2 points **(C)** bruise 2 points **(D)** bruise 3 points **(E)** wound 4 points **(F)** wound 6 points **(G)** wound 6 points **(H)** wound 6 points **(I)** wound 6 points.

**Figure 2:**
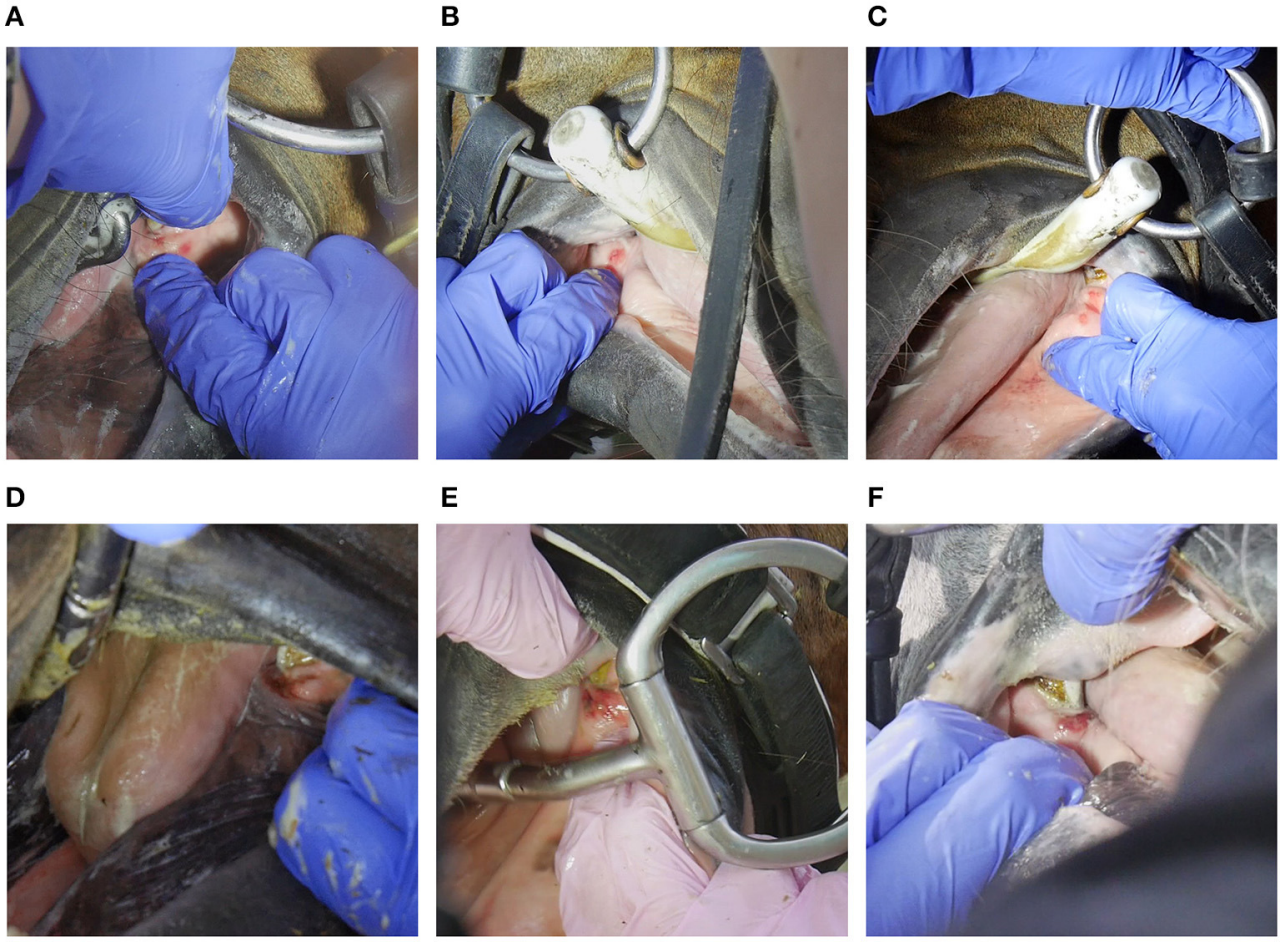
Bit-related bar lesions; **(A)** bruise 1 point **(B)** bruise 2 points **(C)** bruise 3 points **(D)** wound 4 points **(E)** wound 6 points **(F)** wound 6 points.

According to Finnish riding competition rules, horses must be bridled in the competition venue for safety reasons, so the majority of horses were examined with their bridle on. A good view and a reliable examination result was achieved when the noseband and curb chain, if present, were unlatched, allowing the examiner to open the horse's mouth. Fingers were used to lift the bit to achieve better visibility (Figures 1A,C,F, 2A–D). In other regards, the examination was similar to that conducted on Finnish trotters in a previous study and described in Tuomola et al. (11).

### Data Collection

**Figure 3:**
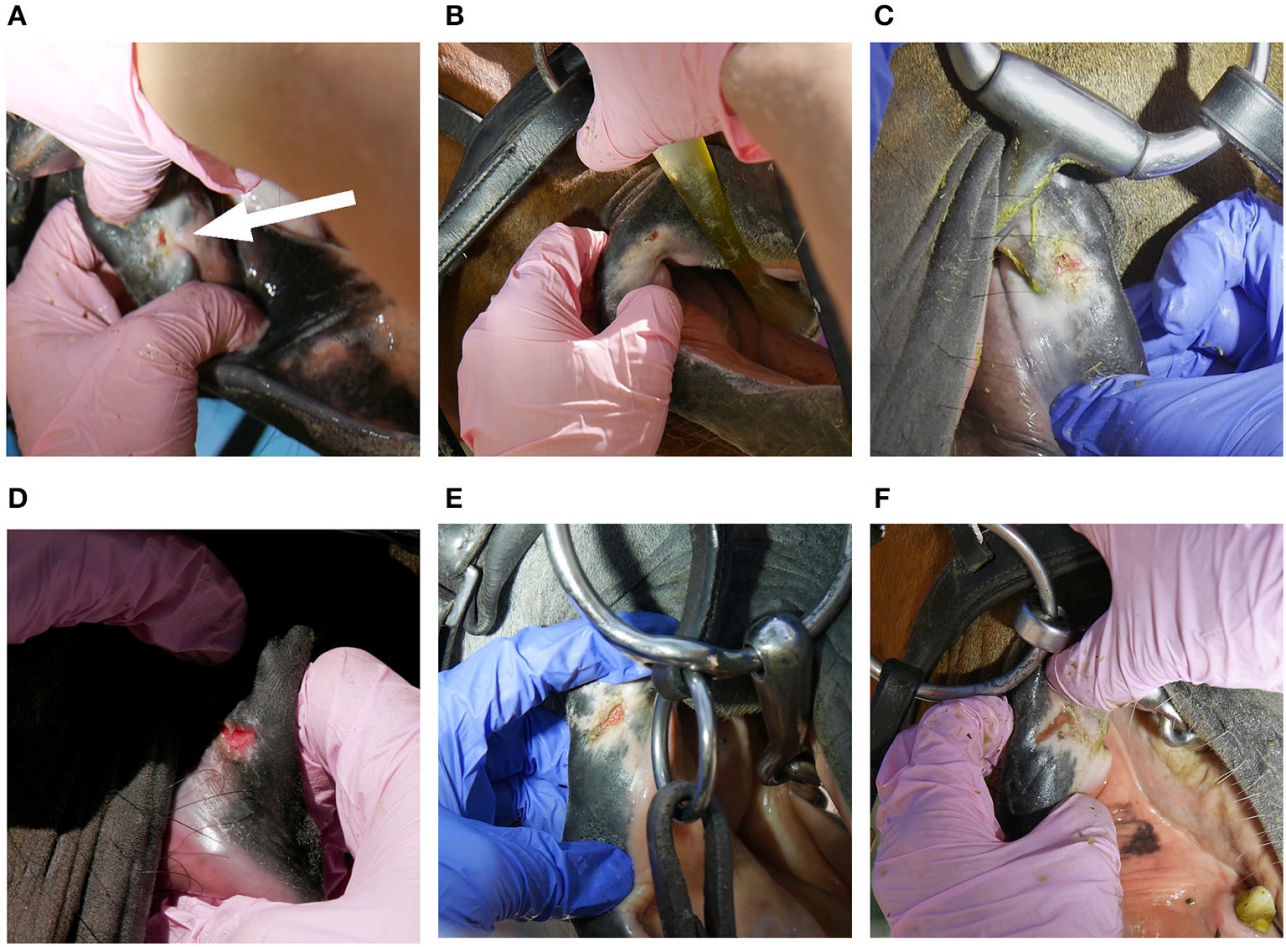
Bit-related outer lip commissures lesions; **(A)** wound 4 points, Arrow: abnormal appearance of lip commissure due to old bit-related lesion. **(B)** Wound 4 points **(C)** wound 4 points **(D)** wound 6 points **(E)** wound 6 points **(F)** wound 6 points.

**Figure 4:**
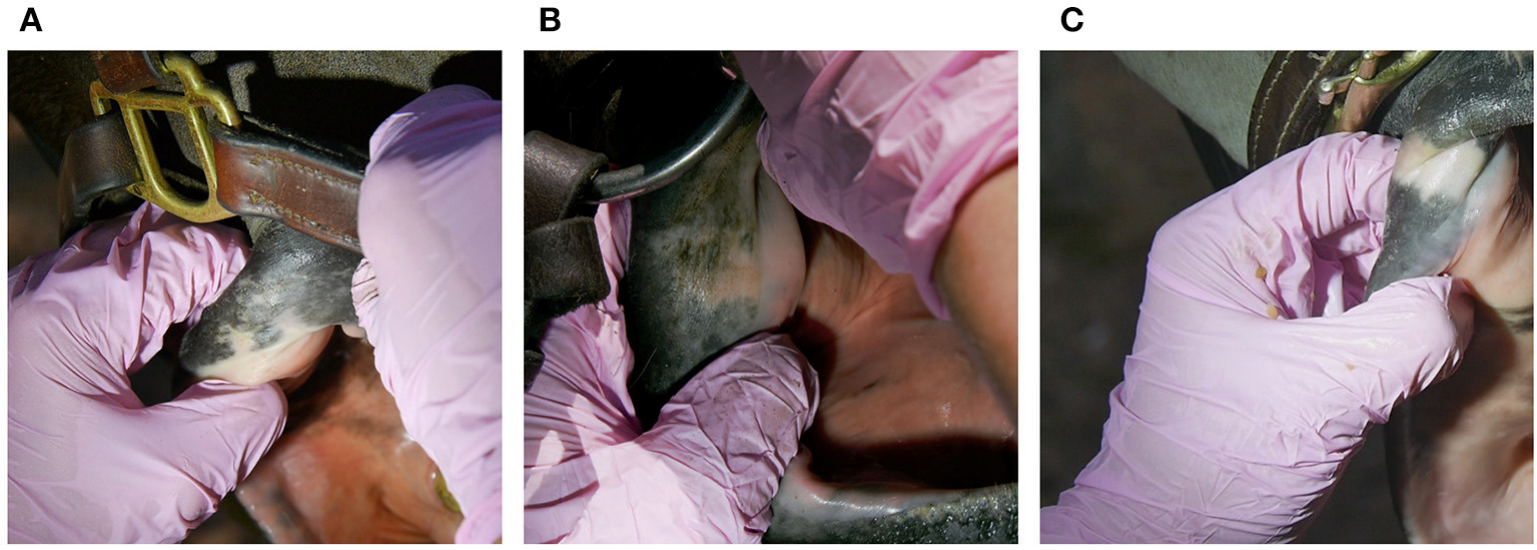
Various degrees of pigment loss on outer lip commissures **(A–C)**. Prolonged bit pressure or previous inflammation may inhibit melanocyte function and cause depigmentation on areas that should normally be pigmented (22, 23).

The following data were collected on oral lesions. *Lesion location* was identified as inner lip commissure, outer lip commissure, bars of the mandible, buccal area near second upper premolar, tongue, or palate. *Lesion type* was recorded as a bruise (submucosal bleeding, but with the mucosa visually intact) (Figures 1A–D, 2A–C) or a wound (mucosal injury) (Figures 1E–I, 2D–F, 3A–F) (11). We did not collect information on periostitis (bone spurs) in the bars (16, 21) or bit-induced second lower premolar abrasion (16). For each acute lesion, points from 1 to 10 were given based on lesion type, size and depth as described in detail in Tuomola et al. (11). Points were added together to form a lesion score determining the lesion severity status of each horse: A (no acute lesions) 0 points, B (mild lesion status) 1–2 points, C (moderate lesion status) 3–11 points, except horses with 8 points from one single lesion, or D (severe lesion status) over 11 points and horses with 8 points from one single lesion (11) (Supplementary Material 1). Wounds with signs of chronicity were graded as acute if the lesion was visibly red and incompletely healed (Figures 1E,F, 3C,E,F). *Old lesions* were recorded separately as scars, old wounds, old bruises, or depigmentation of the outer lip commissures (Figures 4A–C).

Other recorded variables were breed (warmblood, coldblood, or pony), sex and age of the horse, and competition level described as jump height in centimeters. The name of the rider (anonymized for the data analysis) and performance at the current event (placing, no placing, or no final result because the horse did not finish the cross-country test) were verified from the SRL online show management database (KIPA).

Variables related to bit were bit type (basic double-jointed, formed double-jointed, single-jointed, unjointed, Myler type, Waterford or other), bit thickness in millimeters (mm), and bit leverage effect (yes/no). For example Baucher, Gag, and Olympia bits were considered to have a leverage effect. Any double-jointed bit with a distinctive design was considered to be formed. Bit names with distinctive designs or leverage effect are presented in Supplementary Material 2. Due to practical field constraints, bit thickness was measured adjacent to the bit ring with a vernier caliper for only 186 of the examined horses.

Variables related to noseband were noseband type (Cavesson, Cavesson with flash, Mexican, Micklem-type, Drop, or PS of Sweden High Jump or similar) and the presence of a lower noseband (yes/no). Other noseband types apart from Cavesson were considered to have a lower noseband.

### Data Analysis

Data were analyzed statistically using Stata IC version 16 (Stata Corporation, Texas, US). For risk factor analysis, lesion severity categories A–D were merged into two categories: AB (no lesions or mild lesion status) (0–2 points) and CD (moderate or severe lesion status) (over 2 points). Three horse age categories (4–7, 8–12, or 13–19 years), three bit thickness categories (thin bits 10–13 mm, middle-sized bits 14–17 mm, or thick bits 18–22 mm), and three competition level categories (60–80, 90–95, or 100–120 cm) were created. Only ten horses competed at the international level (CCN2^*^ or CCN3^*^ with jump height 115 or 120 cm), and they were merged with the competition level category 110–120 cm. For logistic regression analysis, Myler type bits (*n* = 8) and Waterford bits (*n* = 7) were included in the group “other bit,” as were Bombers 2½ cable bits (*n* = 1), Bombers single-jointed lock-up bits (*n* = 1), and bits without a type category (*n* = 1).

Univariable analyses of the associations between all potential risk factors and the outcome variable of interest (no lesions/mild lesion status vs. moderate/severe lesion status) were first evaluated using Pearson Chi-square or Fisher's exact tests (bit type) (Table 1). Relationships between potential risk factors (multicollinearity) were evaluated by pairwise associations. Breed or sex were not associated with bit type or bit thickness. Bit type and bit thickness were associated (*P* < 0.001), and only bit thickness was entered into the model as it was significantly associated with moderate/severe lesion status (Table 1). Breed and sex were associated so that the majority of stallions (11/13) were coldbloods. Stallions were therefore excluded from the logistic regression analysis. Bit thickness was not measured for all horses (*n* = 22) so the logistic regression analysis was performed with 174 horses. These horses were ridden by 159 individual riders, thus the rider effect could not be reliably analyzed. Biologically relevant interactions were tested and none were found.

**Table 1:** Event horses (*N* = 208) and their risk factors for moderate or severe oral lesion status.

**Variable**	**Category**	***n***	**Moderate/severe lesion status horses**	**% (95% CI)**	***P*-value**
Breed					0.003
	Warmblood	127	37	29 (21–37)	
	Coldblood	52	24	46 (32–60)	
	Pony	29	3	10 (0–21)	
Sex					0.01
	Mare	75	29	39 (28–50)	
	Stallion	13	7	54 (27–81)	
	Gelding	120	28	23 (15–31)	
Age (years)					0.7
	4–7	39	10	26 (12–40)	
	8–12	106	34	32 (23–41)	
	13–19	63	20	32 (20–44)	
Noseband type					0.4
	Cavesson	33	10	30 (14–46)	
	Cavesson with flash	63	19	30 (19–41)	
	Micklem	32	6	19 (5–33)	
	Mexican	43	17	40 (25–55)	
	Drop	16	5	31 (19–43)	
	PS of Sweden high jump	15	7	47 (22–72)	
	Missing value	6			
Lower noseband					> 0.9
	Yes	169	54	32 (25–39)	
	No	33	10	30 (14–36)	
	Missing value	6			
Bit type					0.2
	Double-jointed basic	78	17	22 (13–31)	
	Double-jointed formed	52	17	33 (20–46)	
	Single-jointed	40	16	40 (25–35)	
	Unjointed	20	9	45 (23–67)	
	Myler type	8	3	38 (4–72)	
	Waterford	7	2	29 (0–63)	
	Other	3	0	0	
Bit thickness (mm)					0.007
	10–13	38	16	42 (26–58)	
	14–17	107	23	22 (14–30)	
	18–22	41	18	44 (29–59)	
	Missing value	22			
Leverage					0.9
	Yes	57	18	32 (20–40)	
	No	150	46	31 (24–38)	
	Missing value	1			
Competition level (cm)					0.9
	60–80	103	30	29 (20–38)	
	90–95	62	20	32 (20–44)	
	100–120	43	14	33 (19–47)	
Placement					0.8
	Placement	65	18	28 (17–39)	
	No placement	122	39	32 (24–40)	
	No result	21	7	33 (13–53)	

*The results of the univariable analysis in Pearson Chi-square test. Bit type was tested with Fisher's exact test. All predictors were tested against the outcome: no lesions or mild lesion status vs. moderate or severe lesion status. Thirty-one percent of all horses had moderate/severe lesion status*.

The logistic regression model (Model I) was built with manual stepwise backward and forward procedures and explanatory variables were eliminated until all remaining variables had an association with moderate/severe lesion status with a *P* ≤ 0.05. At each step, the removed variables were evaluated for confounding effects by checking whether the coefficients for the remaining variables changed substantially. The Model I containing breed, sex, and bit thickness was evaluated using tests for sensitivity, specificity, ROC curve (Receiver Operating Characteristic) and goodness of fit and visually from the graphs of the residuals, leverage, and delta-betas per covariate pattern.

Because bit thickness was associated with bit type, we wanted to analyze horses wearing the same bit type and as a consequence double-jointed bitted horses (*n* = 110) were analyzed in a separate model (Model II) including breed, sex and bit thickness. The model building process and evaluation were performed as in Model I.

Associations between lesion location and bit type were analyzed with Fisher's exact test. A *P* < 0.05 was considered statistically significant in all analyses.

## Results

The study included 208 horses: 127 warmbloods (predominantly part-breds), 52 coldblooded riding horses (49 of which were Finnhorses), and 29 ponies of various breeds. Sex distribution was 120 geldings, 75 mares, and 13 stallions. Ages ranged from 4 to 19 years (Median 10, Mean 10.7, SD 3.5). The majority of the horses competed at jump height level 60–80 cm (Table 1).

### Oral Lesions

Of all horses, 52% (CI 45–59%) (109/208) had acute oral lesions in the bit area. Lesion status was no lesions for 48% (CI 41–55%) (99/208), mild for 22% (CI 16–28%) (45/208), moderate for 26% (CI 20–32%) (55/208), and severe for 4% (CI 1–7%) (9/208) of the horses. The results of the univariable analysis are presented in Table 1.

**Figure 5:**
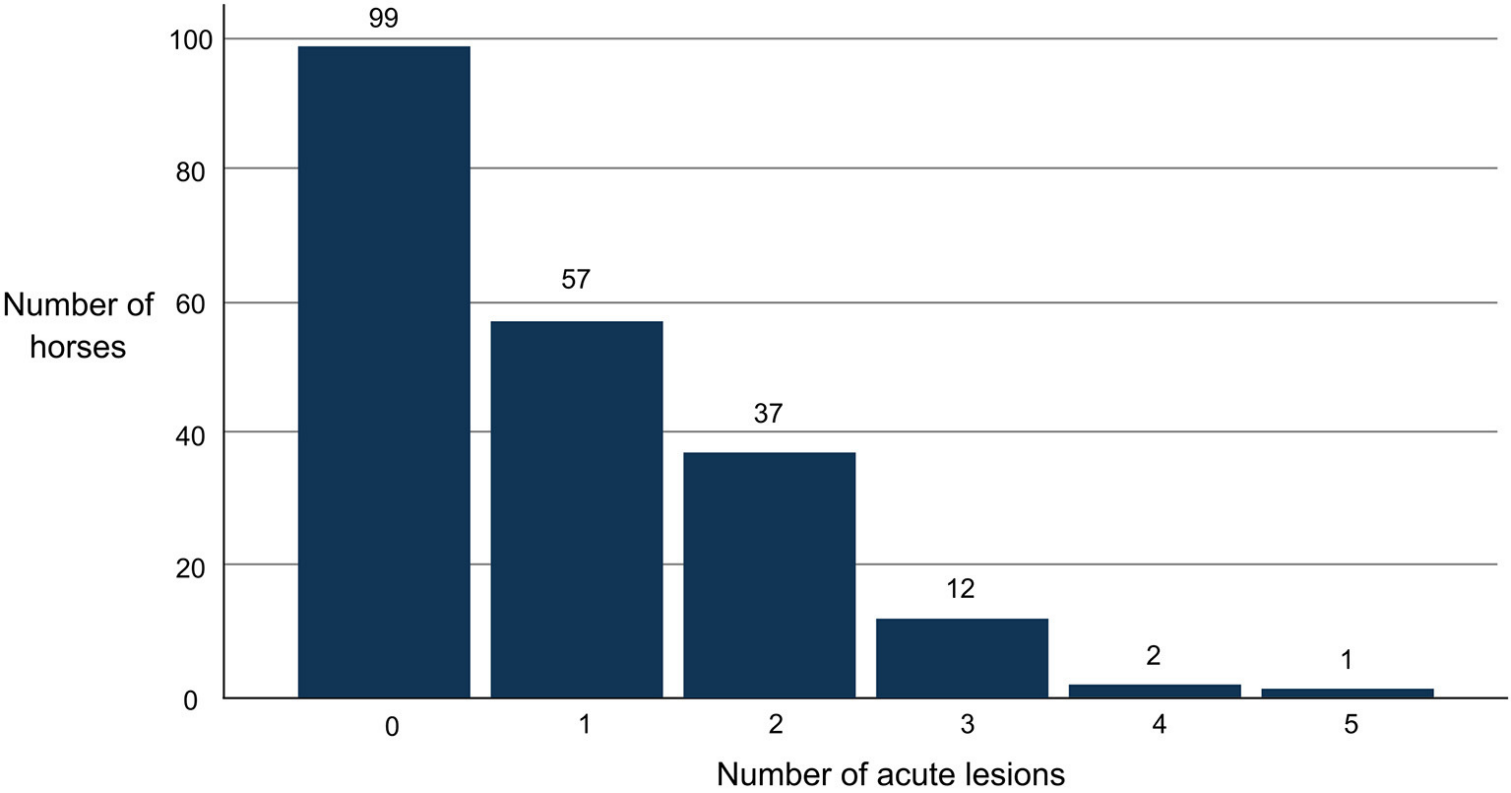
Number of acute bit-related lesions on event horses (*N* = 208) after a cross-country test.

**Figure 6:**
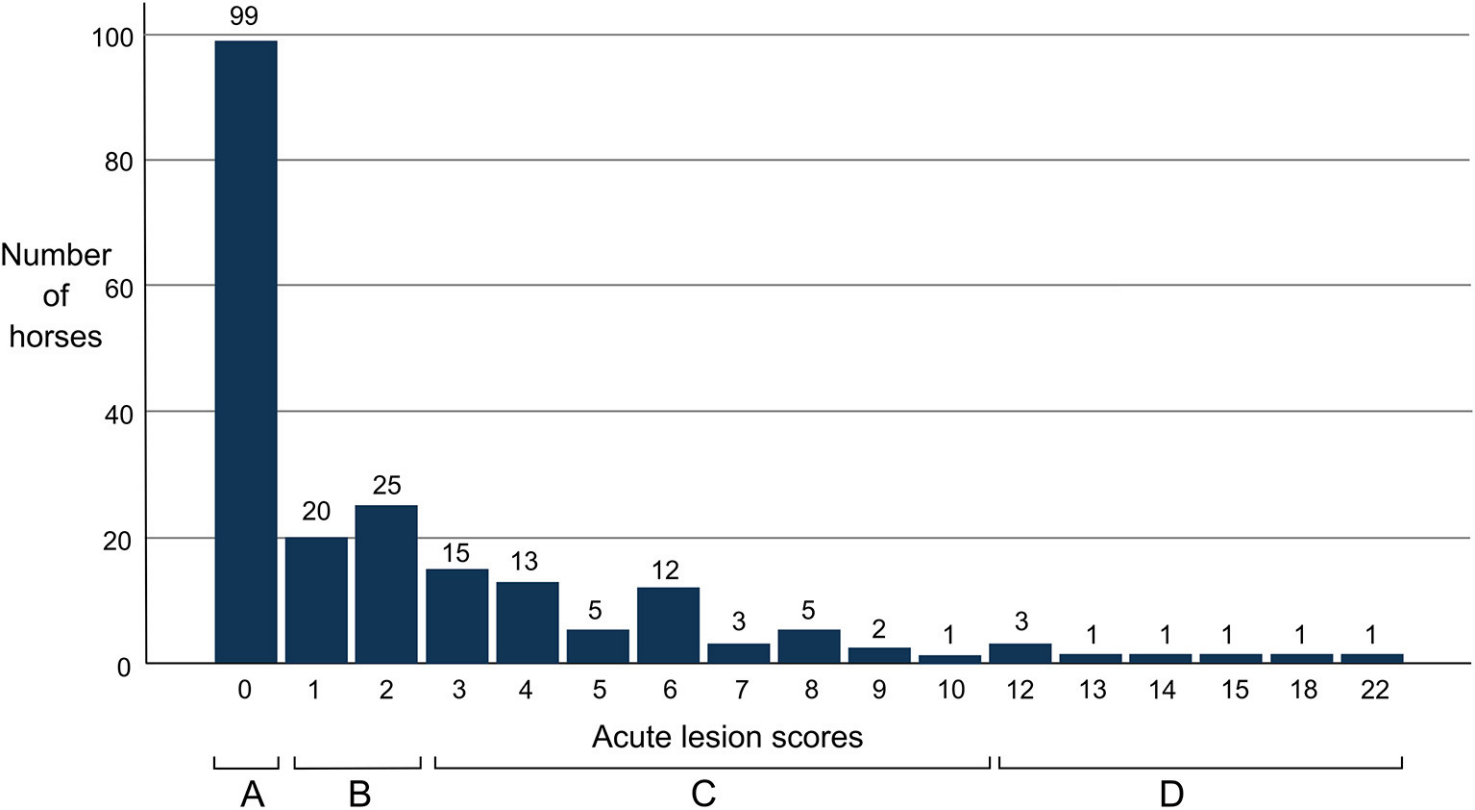
Acute lesion scores (total lesion points) and lesion severity status for event horses (*N* = 208) after a cross-country test.

Bruises were found in 39% of all horses (82/208) and wounds in 19% (40/208). None of the wounds were graded as deep. If lesions were present, there was typically only one. The highest number of acute lesions observed in an individual horse was five (Figure 5). One horse had blood inside the mouth. This horse also had the highest lesion score (total points) 22 (Figure 6).

Old lesions were not included in logistic regression analysis, but it is worth noting that depigmentation of the outer lip commissures was observed in 52% (108/208) (Figures 4A–C), old wounds in 10% (20/208), old bruises in 5% (10/208), and old scars in 3% (6/208) of the horses.

### Lesion location and Bit Type

The inner lip commissure was the most common lesion location, with 39% (81/208) of horses having lesions in this area (Figures 1A–I). Of all horses, 12% (24/208) had lesions in the bars (Figures 2A–F), 9% (18/208) in the outer lip commissures (Figures 3A–F) and 6% (12/208) in the buccal area near the first upper cheek teeth. Only two horses had tongue lesions, located at the lateral edges. No lesions were present on the palate. The double-jointed bit was the most common bit type, present in 65% of the horses. Bar lesions were more common in horses with unjointed bits (40%, 8/20) than with basic double-jointed (10%, 5/52), formed double-jointed (8%, 6/78) or single-jointed bits (5%, 2/40) (Fisher's exact test, *P* = 0.002). Six unjointed bits had ports, and three horses wearing ported unjointed bits had bar lesions. Three of the Myler-type bitted horses (3/8) and none of the Waterford bitted horses (0/7) had bar lesions, but such a small subsample size does not allow for definitive conclusions. Bit type was unassociated with outer lip (Fisher's exact test, *P* = 0.5) or inner lip commissure lesions (Fisher's exact test, *P* = 0.8).

### Logistic Regression Model

Model I containing breed, sex, and bit thickness was statistically significant (*N* = 174, χ^2^ 26.0, *P* < 0.001) indicating that the model was able to distinguish between lesion status AB and CD. Results of the Model I, in Table 2, are presented as odd ratios (OR) and with a 95% confidence interval (CI). Model sensitivity was 37% and specificity was 85%. The model correctly classified 71% of the cases. The area under the ROC curve was 73% (CI 65–80%, *P* < 0.001). Pearson's goodness of fit test supported the model (χ^2^ 10.4, *P* = 0.5).

**Table 2:** Event horses (*N* = 174) and their risk factors for moderate or severe (CD) oral lesion status vs. no lesion or mild lesion (AB) status in the bit area.

**Variable**	**Category**	***n***	**Moderate/severe lesion status horses (%)**	**OR**	**95% CI**	***P*-value**
Breed						0.02
	Warmblood	109	34 (31)	Reference		
	Coldblood	39	15 (39)	2.0	0.88–4.7	0.1
	Pony	26	2 (8)	0.2	0.04–0.87	0.03
Sex						0.03
	Gelding	106	25 (24)	Reference		
	Mare	68	26 (38)	2.2	1.1–4.5	
Bit thickness (mm)						0.003
	14–17	102	20 (20)	Reference		
	10–13	34	14 (41)	3.5	1.4–8.7	0.007
	18–22	38	17 (45)	3.4	1.5–8.0	0.004

*The results of the logistic regression analysis for the Model I containing breed, sex, and bit thickness. Thirty-one percent of all horses in the study had moderate/severe lesion status*.

Horses wearing thin (OR 3.5, CI 1.4–8.7) or thick (OR 3.4, CI 1.4–8.0) bits had a higher risk of moderate/severe lesion status than horses wearing middle-sized bits (reference group) (*P* = 0.003). However, bit thickness was associated with bit type so that double-jointed bits were mostly middle-sized and unjointed bits were mostly thick (*P* < 0.001) (Table 3). Moderate/severe lesion status was observed less in horses wearing basic double-jointed bits, but the result was statistically non-significant.

**Table 3:** Bit type and bit thickness were associated (*P* < 0.001).

**Bit type**	***n***	**10–13 mm (%)**	**14–17 mm (%)**	**18–22 mm (%)**
Double-jointed basic	66	11 (17)	45 (68)	10 (15)
Double-jointed formed	49	6 (12)	38 (78)	5 (10)
Single-jointed	37	9 (24)	16 (43)	12 (32)
Unjointed	17	3 (18)	2 (12)	12 (71)
Other	17	9 (53)	6 (35)	2 (12)

*Some bit types are only available in certain thicknesses. Double-jointed bits were mostly middle-sized and unjointed bits were mostly thick (n = 186)*.

Breed was associated with moderate/severe lesion status (*P* = 0.02). The risk of moderate/severe lesion status was lower for ponies (OR 0.2, CI 0.04–0.87) than warmbloods (reference group) and coldbloods (OR 2.0, CI 0.88–4.7). The difference between coldbloods and warmbloods was not statistically significant. Mares were at higher risk (OR 2.2, CI 1.1–4.5) than geldings (reference group) (*P* = 0.03) (Table 2).

In Model II, only horses wearing various double-jointed bits (*n* = 110) were analyzed. Horses wearing either thin (*n* = 13, OR 4.3, CI 1.3–14) or thick (*n* = 17, OR 4.5, CI 1.2–17) bits had a higher risk of moderate/severe lesion status than horses wearing middle-sized (14–17 mm) bits (*n* = 80) (*P* = 0.01). In Model II sex (*P* = 0.2) and breed (*P* = 0.09) were not significantly associated with moderate/severe lesion status.

### Horse Age, Noseband, Bit Leverage, Competition Level and Performance

All horses wore a noseband, and 84% (169/202) of horses wore an additional lower noseband. Noseband type, presence of lower noseband, bit leverage, horse age, competition level or competition performance (placement, no placement, or no result) were not associated with moderate/severe lesion status (Table 1).

## Discussion

Acute lesions in the bit area were observed in 52% of the event horses after a cross-country test. Lesion status was moderate for 26% of the horses, severe for 4% of the horses. Horses wearing thin or thick bits, mares, and horse breeds were at higher risk of moderate or severe oral lesion status in the bit area. Horses wearing unjointed bits were at higher risk of bar lesions than horses wearing jointed bits.

This study has certain limitations. First, we did not have control over the bit types as the study was executed on a competition setting where the bit selection is made by the participants. The examination was voluntary. Nevertheless, the participation rate for the study (95%) was very high, and the sample represents 25% (208/831) of the Finnish event horses competing in 2018–2019. Second, the horses had competed in dressage and show jumping the day before. If some lesions had occurred during those performances or from earlier training and were still exhibiting as acute lesions, they could have been unrelated to but possibly aggravated by the bit type worn during the cross-country phase. Third, noseband tightness was not measured to minimize inconvenience to the competitors. Noseband tightness has been previously linked with outer lip commissure lesions (13). However, noseband type and the presence of a lower noseband were recorded and we did not find evidence of an association between noseband and moderate/severe lesion status. Finally, the small sample size of coldbloods and other than double-jointed bits may prevent detection of their potential risk regarding moderate/severe lesion status (type II error).

Even though a high occurrence of oral lesions were found in this post-competition sample of Finnish event horses, the occurrence was lower than in trotters in Finland (84%) and Sweden (88%) (11, 14). The current study results are in accordance with bit-related lesions observed in Icelandic horses in 2012 (60%) and 2016 (43%) (9, 10) but differ from results from 2014 when lesion prevalence was 33% (10). The proportion of horses with severe lesion status was smaller in event horses (4%) than in trotters (20%) (11). Likewise, moderate lesion status was less common in event horses (26%) than among trotters (43%), but mild lesion status was as common in event horses (22%) as in trotters (21%) (11). Severe lesions were reported in 8% of Icelandic horses, but this result cannot be compared to the present study due to different grading systems (9). In the Icelandic study a mucosal ulcer larger than 1 cm was graded as severe (9) in contrast to the present study where an ulcer of that size was graded as moderate if it was not deep and not reaching 3 cm in diameter. In the present study, no bleeding was observed outside the mouth, and blood was observed inside the mouth in one horse (0.5%), which was less than in trotters (12%) (11). These differences in lesion severity status and blood occurrence might be explained by differences between the two sports. For example in Finland, 16 horses may compete simultaneously in harness racing, in contrast to eventing, where the horse is performing alone. Close proximity of unfamiliar horses in harness racing may increase anxiety in horses (24). Furthermore, the driver is able to lean fully backwards in the sulky while supporting the feet against the footrests. Sudden forceful or high rein tension due to the above mentioned reasons may predispose trotters to oral trauma.

Bruises were more common than wounds. The inner lip commissure was the most common lesion location, corresponding to findings in a previous study of trotters (11). In the present study no association was found between inner lip commissure or buccal lesions and bit type in contrast to the study where Icelandic horses competing with snaffle bits (*n* = 26) had higher risk of buccal lesions (62%) (buccal lesions in that study included inner lip commisure lesions) than horses competing with curb bits with a port (*n* = 45) 13% (9). Outer lip commissure lesions were observed in 9% of the horses. This finding is similar to studies of Danish riding horses (9%, *N* = 3,143) and Finnish trotters (6%, *N* = 264) (11, 13). Various degrees of depigmentation, indicating prolonged bit pressure or previous inflammation (22, 23), was more common in event horses (52%) than in trotters (10%) (11).

Bar lesions were observed in 12% of the horses. Horses wearing unjointed bits had more bar lesions mesial to second lower premolar teeth than did other horses, which aligns with observations concerning trotters (15). When pressure is applied to the bit, mucosal tissue may get pressed and pinched or lacerated between the bit and the mesial surface of the second lower premolar (25). In Icelandic horses (*n* = 77), bar lesions increased from 8 to 31% during competition and were associated with using curb bits (unjointed and jointed) with ports (9). In trotters, bar lesions were observed in 26% after a race (11). In a study of ridden and non-ridden horses (70 riding horses and ponies, 23 riding horses examined after 5 weeks of rest and after 7 weeks of work, and 20 broodmares), none had bar ulcerations (17). Therefore, it is possible that, in addition to unjointed bit types, factors relating to competitions or comparable training sessions increase the occurrence of bar lesions.

Horses competing with bits categorized as either thin or thick were at higher risk of moderate and severe lesions. Several mechanisms can explain this finding. First, thin bits may cause increased pressure on a relatively small area. One aspect of the mouthpiece's severity is its inverse relation to its diameter (26) as pressure is the amount of force applied to a certain area (*P* = F/A) (27). Second, the distance between the maxilla and the mandible varies individually, ranging between 25 and 43 mm in horses and 25 and 39 mm in ponies (28). This distance was not measured in the current study, making it difficult to estimate whether the recorded bit was too thick to fit the individual. It has been suggested that thick bits may cause more discomfort in horses with small oral cavities (29). Third, double-jointed bits were overrepresented in the middle-sized bit category, while the thin and thick bit categories included relatively more single-jointed and unjointed bits meaning that thickness is a feature of a particular bit type. However, performing a more narrow analysis of horses with various double-jointed bits showed that middle-sized bits were still associated with a lower moderate/severe lesion status risk. Finally, according to the authors' perceptions, 14–17-mm double-jointed bits are commonly used for riding in Finland. It is plausible that riders revert to other bit types or sizes than the commonly used 14–17-mm double-jointed bit when their horses show conflict behavior or reduced rideability, which may be related to increased rein tension (30–32). The way that bit thickness was associated with lesion severity was surprising and needs further verification in a larger sample of horses.

Horse breed and sex were associated with moderate/severe lesion status risk. The risk was lower for ponies than for warmbloods and coldbloods. In previous studies, ponies have exhibited less oral lesions in riding disciplines and harness racing (13, 15, 17). Mares were at higher risk of moderate/severe lesion status than geldings. This finding is in line with that of trotters (15). Different rein tension resulting from differences in horse handling (17, 33), education level (34), pain sensation (35), anxiousness (36, 37), or excitability (37) is a possible mechanism behind these results for mares and ponies. Ponies are typically ridden by children, who have less physical strength to apply to rein tension than adults (17). As for mares, literature suggests that sex-based stereotypes may compromise mare welfare if their behavior is interpreted arising from gendered beliefs or if pain-related behaviors are ignored (33). For both subgroups, differences in mucosal wound susceptibility or healing capacity cannot yet be ruled out, as wound healing is faster in ponies than in horses (38) and, in humans, oral mucosal wound healing capacity is slower in women than in men (39).

Other variables were unassociated with moderate/severe lesion status. Most event horses in our present study competed nationally at a low difficulty level (60–80 cm). Oral lesions were unconnected to competition level, in contrast to a Danish study where the number of commissure lesions increased with competition level (13).

Good performance is still often held as an indicator of good welfare (40). Evidence of association between lesion status and competition placement was not found in the present study and a previous study (15) also showed no association with race performance. Therefore, well performing horses are not necessarily free from welfare concerns.

## Conclusions

If the size of the horse's oral cavity is not known, it is advisable to choose a middle-sized jointed bit and pay attention to handling of mares and warmblooded and coldblooded horses since this study suggests that horses wearing thin or thick bits as well as mares, warmblooded and coldblooded horses had an elevated risk of moderate or severe oral lesion status compared with horses wearing middle-sized bits, geldings, and ponies. Furthermore, horses wearing unjointed bits were at higher risk to get bar lesions than horses wearing jointed bits.

We encourage adopting bit area monitoring as a new routine by horse handlers and as a welfare measure by competition organizers for randomly drawn horses since oral lesions in the bit area were common after a competition even though no external bleeding was observed. In riding horses bit-related lesions can be monitored in competition environment with systematic oral examination by using a headlamp, unlatching nosebands and possible curb chain, and lifting the bit while examining the bars of the mandible.

## Data Availability Statement

The raw data supporting the conclusions of this article will be made available by the authors, without undue reservation.

## Ethics Statement

The animal study was reviewed and approved by University of Helsinki Viikki Campus Research Ethics Committee. Written informed consent for participation was not obtained from the owners because The participants received information concerning the study upon online registration for competitions and from an information leaflet pertaining to the study handed out on the 1st day when they checked in at the competition office. The examination was voluntary for the participants. Consent for the examination was asked orally due to field conditions in competitions. Anonymity of the riders was maintained.

## Author Contributions

KT contributed to the study design, performed the oral examinations, data collection and analyses, and preparation of the manuscript. MK–W and KT performed the statistical analysis, have had full access to all data in the study and take responsibility for the integrity of the data, and the accuracy of the data analysis. NM–K recorded all the findings, contributed to data collection, and manuscript preparation. MK–W, AV, and AM contributed to interpreting the results and manuscript preparation. All authors have read and approved the final manuscript.

## Conflict of Interest

The authors declare that the research was conducted in the absence of any commercial or financial relationships that could be construed as a potential conflict of interest.

## Supplementary Material

Supplementary Material 1 Scoring of oral lesions. Estimate lesion size and type. See points at intersection and estimate depth of wound. From each lesion count the points together to form lesion score for the horse. Lesion score determines the severity category of the oral lesion status A (no acute lesions), horses with 0 points; B (mild lesion status), horses with 1–2 points; C (moderate lesion status), horses with 3–11 points, but excluding horses with eight points from one single lesion; and D (severe lesion status), horses with 12 or more points and horses with eight points from one single lesion (11).

Supplementary Material 2 Bit names with distinctive designs or leverage effect.
