# Supplementary material for: Bit-Related Lesions in Event Horses After a Cross-Country Test
Source: Front Vet Sci. 2021 Mar 31;8:651160. doi: 10.3389/fvets.2021.651160 (PMC8044447; doi:10.3389/fvets.2021.651160)
Supplement: Supplementary Material 1 — Scoring of oral lesions. Estimate lesion size and type. See points at intersection and estimate depth of wound. From each lesion count the points together to form lesion score for the horse. Lesion score determines the severity category of the oral lesion status A (no acute lesions), horses with 0 points; B (mild lesion status), horses with 1–2 points; C (moderate lesion status), horses with 3–11 points, but excluding horses with eight points from one single lesion; and D (severe lesion status), horses with 12 or more points and horses with eight points from one single lesion (11). [file Data_Sheet_1.PDF]

LESION SIZE	LESION TYPE		
	Bruise	Wound	Wound: superficial or deep?
< 5 mm	**1**	**2**	if deep, add **+ 2**
≤ 1 cm	**2**	**4**	
< 3 cm	**3**	**6**	
≥ 3 cm	**4**	**8**	
