# Supplementary material for: Bit-Related Lesions in Event Horses After a Cross-Country Test
Source: Front Vet Sci. 2021 Mar 31;8:651160. doi: 10.3389/fvets.2021.651160 (PMC8044447; doi:10.3389/fvets.2021.651160)
Supplement: Supplementary Material 2 — Bit names with distinctive designs or leverage effect. [file Data_Sheet_2.pdf]

The following bit types were considered to have a leverage effect:
Baucher
Bombers 2½ ring
B-ring
Gag
Kimblewick
Neue Schule Jumpers Choice
Neue Schule Tranz Universal
Olympia
Pelham
Pessoa
Neue Schule Turtle Top Beval
Sprenger Dynamic RS Multiring
Stubben Golden Wing
Tom Thumb Butterfly

The following double-jointed bit types with distinctive designs were considered to be formed:
Bombers Colin Miles
Bombers Elliptical Roller
Happymouth
Neue Schule Turtle Tactio
Neue Schule Turtle Top
Rotary bit
Sprenger Dynamic RS
Sprenger KK Ultra
Sprenger Novocontact
Stubben Golden Wing
